# Correction: Pupil diameter differentiates expertise in dental radiography visual search

**DOI:** 10.1371/journal.pone.0240139

**Published:** 2020-09-29

**Authors:** Nora Castner, Tobias Appel, Thérése Eder, Juliane Richter, Katharina Scheiter, Constanze Keutel, Fabian Hüttig, Andrew Duchowski, Enkelejda Kasneci

The ORCID iD is missing for the seventh author, Fabian Hüttig. The author’s ORCID iD is: 0000-0002-7253-5923 (https://orcid.org/0000-0002-7253-5923).
