# Supplementary figures and images for: Pupil diameter differentiates expertise in dental radiography visual search
Source: PLoS One. 2020 May 29;15(5):e0223941. doi: 10.1371/journal.pone.0223941 (PMC7259659; doi:10.1371/journal.pone.0223941)

## Pupil Diameter Size from Baseline for Images

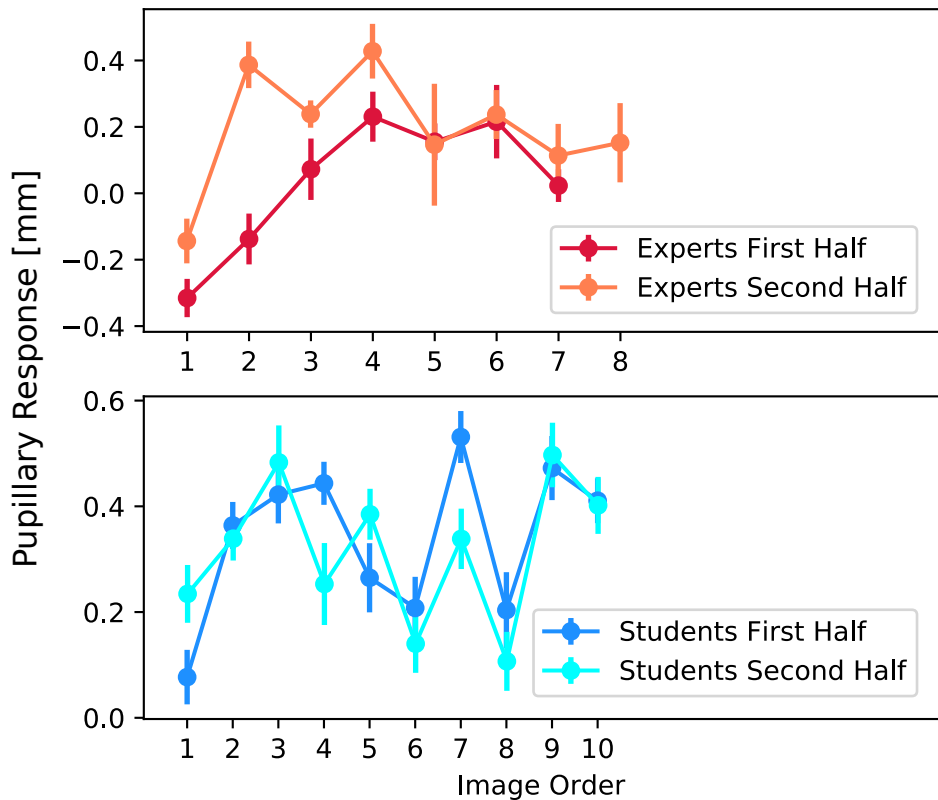

Supplement: S1 Fig — The average pupillary response from baseline for students (blue bars, 20 images total) and experts (red bars, 15 images total) during the first set of OPTs presented and the second set of OPTs presented. Their is no effect in the pupillary response that could be attributed to fatigue during the experiment. (PDF) [file pone.0223941.s001.pdf]
