# Supplementary material for: Pupil diameter differentiates expertise in dental radiography visual search
Source: PLoS One. 2020 May 29;15(5):e0223941. doi: 10.1371/journal.pone.0223941 (PMC7259659; doi:10.1371/journal.pone.0223941)
Supplement: S1 Table — Shows the gaze hits on each anomaly type for both students and experts. For both levels of expertise, the least difficult and intermediate have the most gaze hits. The following are the ambiguous and the most difficult anomalies. Students had overall more gaze hits than experts; however, this may be attributed to the 90 second viewing time they had in comparison to the 45 second viewing time that the experts had. (PDF) [file pone.0223941.s002.pdf]

Table 2. Raw Gaze Count on Anomaly.

Anomaly Type	Less Difficult	Intermediate	More Difficult	Ambiguous
Total	471	448	173	304
Student	312	296	124	202
Expert	159	152	49	102
