# Supplementary material for: Pupil diameter differentiates expertise in dental radiography visual search
Source: PLoS One. 2020 May 29;15(5):e0223941. doi: 10.1371/journal.pone.0223941 (PMC7259659; doi:10.1371/journal.pone.0223941)
Supplement: Attachment — Submitted filename: Response to Reviewers.docx [file pone.0223941.s004.docx]

Dear Dr. Susana Martinez-Conde,

We would like to thank the reviewers for their constructive feedback and suggestions to further improve the manuscript. We are pleased that reviewer #1 feels that all comments have been addressed. In the following, please find reviewer #2’s suggestions in italicized and the changes made to cover these in non-italicized.

**Response to Reviewer #2 Comments:**

Thanks for raising these points, we hope we have addressed them appropriately.

*LINES 21/22: Since the abbreviation “OPT” is not intuitive, “…inspectors of dental panoramic radiographs (OPT)” might suggest that OPT stands for specialists examining radiographs (rather than for the radiographs themselves).*

We thank you for pointing out the clarity issue from the grammar. We have since split the sentence into two to avoid the sentence subject confusion. We have also added the technical term for these dental radiographs, which is where the abbreviation comes from.

*Traditionally the “Introduction” section comes after “Abstract” and before “Material and Methods”. The manuscript, however, contains additional (non-standard) sections (“Background” and “Related work”), which might violate the submission guidelines. I suggest using subheadings, so that all information provided between lines 2 and 162 falls under “Introduction”.*

We would not like to violate the submission guidelines and we have made the background and related work subheadings to the introduction section. Now the introduction consists of three subsections: Background, cognitive load, related work. Paragraph headings in the introduction have also been removed, to avoid confusion as well.

Additionally, we realized that Plos One does not permit footnotes, thus we have removed all footnotes, only incorporating necessary ones into the text.

*There is still significant overlap/redundancy between paragraph 35-45 and paragraph 152-162.*

We have removed the paragraph originally at lines 35-45 and combined a few sentences into the paragraph originally at lines 152-162.

*LINE 71: “… and devote shorter fixation time to an anomaly”. Is this correct? From the provided references, I understand that the experts show a fast initial fixation on the abnormality; do they also devote shorter fixation time to an anomaly than novices?*

In the reference by van der Gijp and colleagues (2017), they found that for detection tasks, experts spent less time fixating on lesions compared to novices for detections tasks. However, they also point out that when the task involves diagnostic reasoning, then the fixation duration is higher than novices.

We have now specified in line 71 (now line 62) that shorter fixation durations for detection tasks.

*LINE 205-206: Please revise the following sentence: “There was unlimited time for the marking phase, and continued with a button click.”*

We have changed the sentence to “There was unlimited time for the marking phase and participants could continue with a button click.”

*LINE 227: “shows four OPT images viewed in the experiment”; should be “shows three OPT….”*

We have change “four” to “three”. Thank you for pointing out this inconsistency from the original version.

*LINE 246-247: Please revise the following sentence: “Certain anomalies inside another and that were highly similar in nature,…”*

We have changed the sentence to “Similar anomalies inside of another, such as (2a,b) in Fig.2.C, were grouped together as one anomaly.” We hope this is more understandable.

*LINE 252: “… an spatial accuracy errors in the gaze.” Should be “… the spatial accuracy errors in the gaze.”*

We have changed “an” to “the”.

*Caption figure 3: “… could incorrectly labeled as blinks. “. Something about the construction of this sentence is not right.*

You are correct, “be” was missing as well as a comma. We have now changed the sentence to “The particular subject in 3a had a high tracking ratio, though many data samples could be incorrectly labeled as blinks.”

*LINES 329-330: “Since baseline data was the two second fixation cross directly before each stimulus…”. Do you mean: “Since baseline data was sampled during the two seconds the fixation cross was displayed….”?*

Yes, and we have rewritten the line based on your suggestion.

*LINES 445-446: Please revise the following sentence: “Whereas bottom-up strategies that student generally employ focus on salient, noticeable images features regardless of relevancy and is less efficient.”*

We have changed the sentence to “Whereas bottom-up strategies that student generally

employ is less efficient, as focus is on salient, noticeable images features, regardless of

relevancy.”

*LINES 499-501: Please revise the following sentence: “Experts, renowned for their streamlined processing abilities, are able to selectively allocate their attention to relevant information and is evident in the pupillary response.”*

We have condensed the sentence to “Experts are able to selectively allocate their attention to relevant information and is evident in the pupillary response”.
